# Association Between Arterial Stiffness and Blood Pressure Progression With Incident Hypertension: A Systematic Review and Meta-Analysis

**DOI:** 10.3389/fcvm.2022.798934

**Published:** 2022-02-11

**Authors:** Alicia Saz-Lara, Rosa María Bruno, Iván Cavero-Redondo, Celia Álvarez-Bueno, Blanca Notario-Pacheco, Vicente Martínez-Vizcaíno

**Affiliations:** ^1^Universidad de Castilla-La Mancha, Health and Social Research Center, Cuenca, Spain; ^2^INSERM U970, Paris Cardiovascular Research Centre-PARCC, Université de Paris, Paris, France; ^3^Pharmacology Unit, Hôpital Européen Georges Pompidou, AP-HP, Paris, France; ^4^Rehabilitation in Health Research Center (CIRES), Universidad de las Américas, Santiago, Chile; ^5^Universidad Politécnica y Artística del Paraguay, Asunción, Paraguay; ^6^Universidad Autónoma de Chile, Facultad de Ciencias de la Salud, Talca, Chile

**Keywords:** incident hypertension, arterial stiffness, pulse wave velocity, systolic blood pressure, diastolic blood pressure

## Abstract

**Background:**

Arterial stiffness is an independent predictor of cardiovascular and all-cause mortality that is classically regarded as a consequence of arterial hypertension. However, a growing number of studies have shown that arterial stiffness is involved in the pathogenesis and prognosis of arterial hypertension. Thus, in this systematic review and meta-analysis, we aimed to assess whether arterial stiffness, as measured by pulse wave velocity, systolic blood pressure and diastolic blood pressure are associated with incident hypertension.

**Methods:**

The Scopus, PubMed, Web of Science and Cochrane Library databases were searched from inception to March 30, 2021. The DerSimonian and Laird method was used to compute pooled relative risk estimates and their respective 95% confidence intervals of association between incident hypertension with pulse wave velocity, systolic blood pressure and diastolic blood pressure.

**Results:**

Our findings provide a synthesis of the evidence supporting that the higher arterial stiffness (RR: 1.09; 95% CIs: 1.05, 1.12), systolic blood pressure (RR: 1.08; 95% CIs: 1.05, 1.10) and diastolic blood pressure (RR: 1.08; 95% CIs: 1.04, 1.12) are associated with incident hypertension in normotensive adult subjects, with similar independent predictive values. However, our results should be interpreted with caution because the meta-analyses performed showed considerable heterogeneity.

**Conclusions:**

Our results showed that higher pulse wave velocity, systolic blood pressure and diastolic blood pressure are associated with incident hypertension. These findings are of clinical importance, supporting arterial stiffness as an additional tool for the prevention of arterial hypertension and being a fundamental component to reduce cardiovascular morbidity and mortality.

**Systematic Review Registration:**

This study was registered in PROSPERO https://www.crd.york.ac.uk/PROSPERO/display_record.php?RecordID=236435 (Registration number: CRD42021236435).

## Introduction

Arterial hypertension is associated with higher cardiovascular morbidity and mortality (1). Previous evidence has shown that both the incidence and prevalence of hypertension increase with age (2), making prevention and the early detection of this condition essential (2, 3), in addition to the need to better understand its etiology (3). Incident hypertension is defined as the first occurrence on any follow-up examination of a systolic blood pressure (SBP) of 140 mm Hg or higher, or a diastolic blood pressure (DBP) of 90 mm Hg or higher, or that the person was taking antihypertensive medication (4). Furthermore, different modifiable lifestyle risk factors for hypertension have been recognized, such as smoking (5), an unhealthy diet (6), physical inactivity (6–8), and overweight or obesity (5, 8).

Arterial stiffness (AS) is one of the earliest detected indicators of both functional and structural changes of the arterial wall and is recognized as a direct and independent predictor of cardiovascular and all-cause mortality (9–12). Carotid-to-femoral pulse wave velocity (cf-PWV) is considered the gold standard technique for the non-invasive measurement of AS (13); recently, simpler techniques such as brachial-to-ankle PWV (ba-PWV) have also been increasingly used (13–15). Early assessment of this subclinical marker of cardiovascular disease (CVD) can provide information on complications that can develop years later, such as hypertension (16).

AS is classically considered a consequence of hypertension, and it is listed by recent ESC/ESH hypertension guidelines among biomarkers of hypertension-associated organ damage (17). However, an increasing number of studies have shown that AS is involved in both the pathogenesis and prognosis of hypertension (2, 18, 19). Previous evidence has established an association between AS and blood pressure (BP) levels, considering higher BP as a major cardiovascular risk factor leading to arterial wall damage (20). Some studies consider this relationship to be bidirectional: elevated BP, established as the sum of mean blood pressure (MAP) and pulse pressure (PP) (20), can cause damage at the vascular level (21), and in turn, the early return of the arterial wave reflection that induces AS causes an higher SBP and a decrease in DBP (14, 18, 22).

Although the association between AS and hypertension has been described in several studies (2, 19, 21), it is unclear whether AS precedes the development of hypertension. Therefore, the aims of this systematic review and meta-analysis were (i) to assess whether AS, as measured by PWV, is associated with incident hypertension; (ii) to assess whether SBP and DBP in the normotensive range are associated with incident hypertension; and (iii) to estimate whether the predictive ability of PWV for incident hypertension is independent of SBP.

## Methods

This systematic review and meta-analysis was reported according to the Meta-analysis of Observational Studies in Epidemiology statement (MOOSE) (23) and performed following the recommendations of the Cochrane Collaboration Handbook (24). This study was registered in PROSPERO (Registration number: CRD42021236435).

### Search Strategy

Systematic searches of the Scopus, PubMed (via MEDLINE), Web of Science and Cochrane Library databases were conducted from their inception to March 30, 2021. To perform the search, the following free terms, combined with Boolean operators, were used following the PICO strategy (population, intervention/exposure, comparison and outcome): “normotensive adults,” “young adults,” “older adults,” “elderly adults,” “pulse wave velocity,” “PWV,” “arterial stiffness,” “aortic stiffness,” “blood pressure,” “systolic blood pressure,” “SBP,” “diastolic blood pressure,” “DBP,” “onset hypertension,” “development hypertension,” and “incident hypertension.” The search strategy in the MEDLINE database is shown in Supplementary Table 1. Furthermore, we searched the reference lists of the included articles, as well as previous systematic reviews or meta-analyses. A final search was performed just before the final analysis to include the most recently published studies.

### Selection Criteria

The inclusion criteria were as follows: (i) population: normotensive subjects older than 18 years; (ii) exposure: arterial stiffness measured by PWV, SBP, and DBP; (iii) outcome: incident hypertension; and (iv) study design: prospective longitudinal design. We excluded (i) review articles, editorials, or case reports and (ii) articles that were not written in English or Spanish.

The literature search and study selection were performed independently by two reviewers (AS-L and IC-R), and disagreements were solved by consensus or with the participation of a third researcher (RM-B).

### Data Extraction and Quality Assessment

The main characteristics of the included studies are summarized in Table 1, which includes information on (1) reference: first author and year of publication, (2) the country in which the study data were collected, (3) length of follow-up, (4) population characteristics: sample size (% women), mean age, disease prevalence, smoking history, (5) type of exposure: PWV (cf-PWV, ba-PWV), SBP, DBP, and baseline levels, and (6) incident hypertension: sample size and percentage of subjects that developed hypertension.

**Table 1:** Characteristics of the included studies.

**References**	**Country**	**Follow-up (years)**	**Population characteristics**			**Exposure (PWV, SBP, DBP)**		**Incident hypertension (*n*, %)**
			**Sample size (*n*, % women)**	**Mean age (years)**	**Smoking history (%)**	**Type of exposure**	**Basal levels (m/s or mmHg ±SD)**	
Najjar et al. (2)	Italy	5	449 (55.2)	53.0 ± 17.0	52.0	cf-PWV	6.9 ± 2.5	105 (34.0)
Satoh et al. (25)	Japan	3	2,278 (0)	46.0 ± 6.0	51.1	ba-PWV	13.0 ± 1.4	151 (6.6)
Takase et al. (26)	Japan	4	2,496 (38.2)	57.4 ± 8.7	25.6	ba-PWV	15.1 ± 2.9	698 (28.0)
						SBP	120.7 ± 12.1	
						DBP	73.4 ± 8.5	
Kaess et al. (21)	United States	4	1,048 (NA)	60.0 ± 9.0	12.0	cf-PWV	10.4 ± 3.8	338 (33.0)
						SBP	128.0 ± 17.0	
						DBP	74.0 ± 10.0	
Tomiyama et al. (27)	Japan	3	1,268 (0)	43.0 ± 8.0	31.0	ba-PWV	12.5 ± 1.3	154 (12.2)
						SBP	120.0 ± 10.0	
						DBP	72.0 ± 9.0	
Zheng et al. (19)	China	2.3	2,153 (NA)	54.0 ± 11.0	31.2	ba-PWV	15.8 ± 3.5	432 (20.1)
Koivistoinen et al. (28)	Finland	4	1,183 (58.0)	38.0 ± 5.0	17.0	ba-PWV	7.9 ± 1.9	88 (7.4)
						SBP	120.0 ± 14.0	
						DBP	75.0 ± 11.0	
Wang et al. (29)	China	2.3	1,607 (68.1)	54.2 ± 7.5	19.9	SBP	125.5 ± 14.0	211 (13.1)
Kario et al. (30)	Japan	10	34,649 (53.6)	44.2 ± 12.2	21.0	SBP	118.7 ± 11.3	13,859 (40.0)
						DBP	70.1 ± 8.9	
Lee et al. (14)	Australia	2.2	10,360 (24.4)	40.2 ± 7.2	30.2	ba-PWV	–	2,000 (19.3)
Jiang et al. (31)	China	2.4	1,849 (68.5)	54.2 ± 7.5	20.1	ba-PWV	15.0 ± 2.8	248 (13.4)
						SBP	123.0 ± 9.8	
Sigiura et al. (32)	Japan	4	7,840 (41.4)	51.0 ± 11.7	24.0	SBP	107.4 ± 12.5	2,608 (33.3)

*Data are shown as mean ± standard deviation (SD); ba-PWV, brachial to ankle pulse wave velocity; cf-PWV, carotid to femoral pulse wave velocity; DBP, diastolic blood pressure; NA, not available; PWV, pulse wave velocity; SBP, systolic blood pressure*.

The Quality Assessment Tool for Observational Cohort and Cross-Sectional Studies from the United States National Institute of Health National Heart, Lung, and Blood Institute (33) was used to assess the risk of bias according to the following domains: quality of the research question, reporting of the population definition, participation rate, recruitment, sample size, appropriateness of statistical analyses, timeframe for associations, exposure levels, ascertainment of the exposure, appropriateness of the outcome measured, outcome blinding of researchers, loss to follow-up, and confounding variables. The overall bias of each study was considered “good” if most criteria were met and with a low risk of bias; “fair” if some criteria were met and with a moderate risk of bias; or “poor” if few criteria were met and with a high risk of bias.

Data extraction and quality assessment were conducted by two independent reviewers (AS-L and IC-R), and inconsistencies were resolved by consensus or with the participation of a third researcher (RM-B).

### Data Synthesis and Statistical Analysis

The DerSimonian and Laird random effects method (34) was used to compute pooled estimates of relative risk (RR) and their respective 95% confidence intervals (95% CIs) for the risk of incident hypertension associated with PWV, SBP or DBP. In addition, a predictive model plot was used to estimate the risk of incident hypertension in those studies with two markers (PWV and SBP). Meta-analyses required at least five studies in each exposure group (35). Heterogeneity was assessed using the *I*^2^ statistic, which ranges from 0 to 100%. According to the *I*^2^ values, heterogeneity was considered not important (0 to 30%), moderate (30 to 60%), substantial (60 to 75%), or considerable (75 to 100%) (36). The corresponding *p-value*s were also considered.

Sensitivity analysis (systematic reanalysis removing studies one at a time) was conducted to assess the robustness of the summary estimates. Subgroup analyses were performed according to the type of PWV (cf-PWV or ba-PWV). Random-effects meta-regressions were used to assess whether mean age, percentage of women, smoking history or follow-up time, as continuous variables, modified the association between the risk of incident hypertension with PWV, SBP or DBP. Finally, publication bias was evaluated using Egger's regression asymmetry test (37). A level of <0.10 was used to determine whether publication bias was present.

Statistical analyses were performed using STATA SE software, version 15 (StataCorp, College Station, TX, USA).

## Results

### Baseline Characteristics

**Figure 1:**
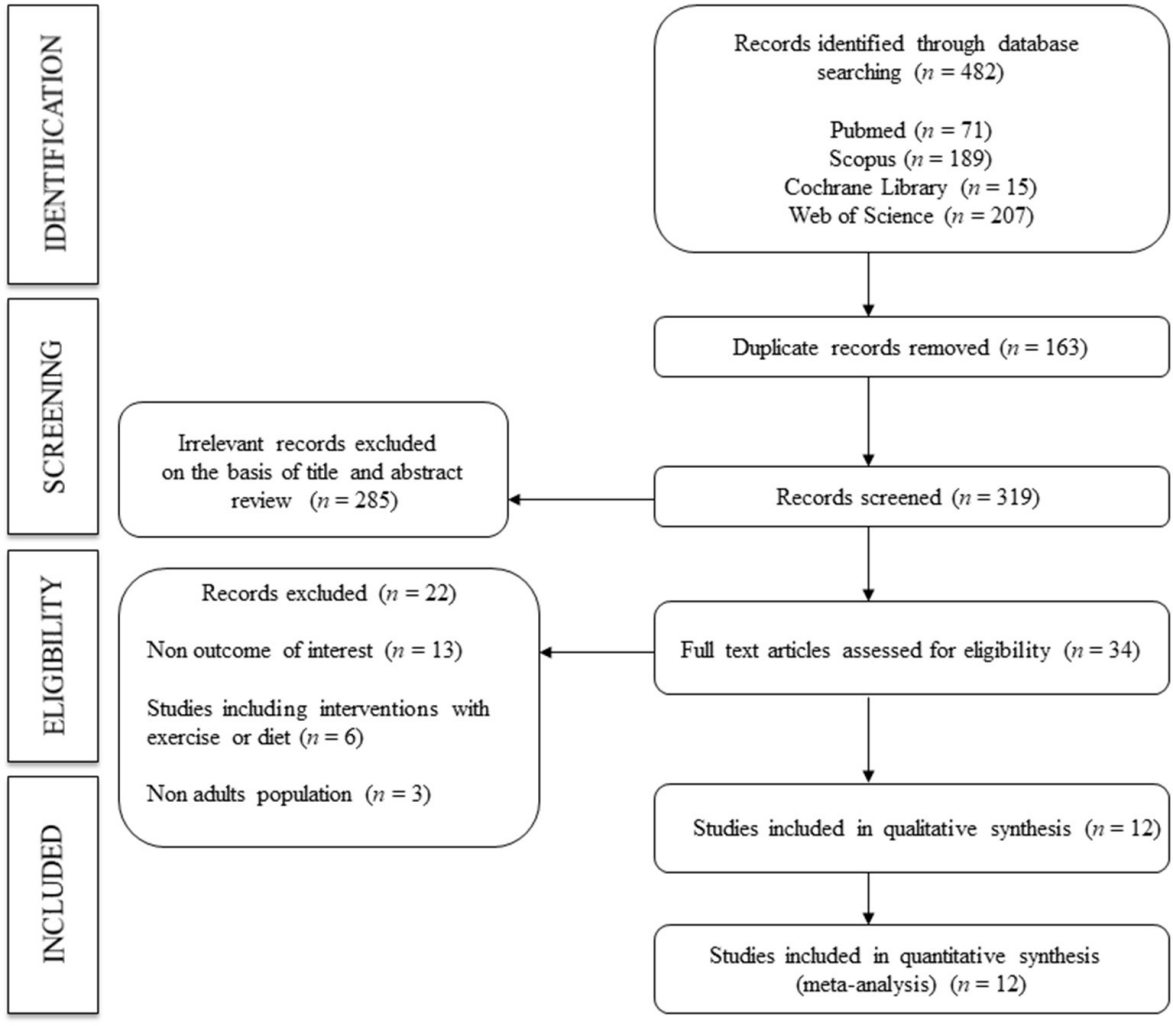
Flowchart: Search strategy.

A total of 12 studies (2, 14, 19, 21, 25–32) were included in the systematic review and meta-analysis (Figure 1). All the included studies were prospective longitudinal studies (follow-up time range: 2 to 10 years) conducted in six countries: five in Japan (25–27, 30, 32), three in China (19, 29, 31), one in Australia (14), one in Finland (28), one in Italy (2), and one in the United States (21). Records were published between 2008 and 2020 and included a total of 66,180 normotensive subjects (aged 38.0 to 60.0 years). Regarding the type of exposure for incident hypertension, nine studies reported on PWV (seven for ba-PWV and two for cf-PWV), eight reported on SBP and five reported on DBP. In addition, five studies (21, 26–28, 31) included two markers (PWV and SBP) and were thus used to calculate the predictive risk value of incident hypertension. Finally, of the total of subjects included, 20,892 (31.6%) developed hypertension during a follow-up period of 2.2 to 10 years (Table 1).

### Quality Assessment and Potential Bias

The overall risk of bias for studies examining the association between PWV and incident hypertension was low in 33.3%, moderate in 55.6%, and high in 11.1% of the included studies (Supplementary Table 2). The overall risk of bias for studies examining the association between SBP and incident hypertension was low in 12.5%, moderate in 75.0%, and high in 12.5% of the included studies (Supplementary Table 3). Finally, the overall risk of bias for studies examining the association between DBP and incident hypertension was low in 20.0%, moderate in 60.0%, and high in 20.0% of the included studies (Supplementary Table 4). For all exposures, we were able to identify three main reasons for a high risk of bias: (i) the follow-up time was not long enough (more than 4 years) (38) to establish an association between the exposure and outcome; (ii) the exposure measurement was assessed only once during follow-up; and (iii) loss to follow-up was >20.0% or the studies did not provide this information. In addition, none of the studies provided information on whether the researchers were blinded to the exposure status of the participants.

### Association Between Arterial Stiffness and Incident Hypertension

**Figure 2:**
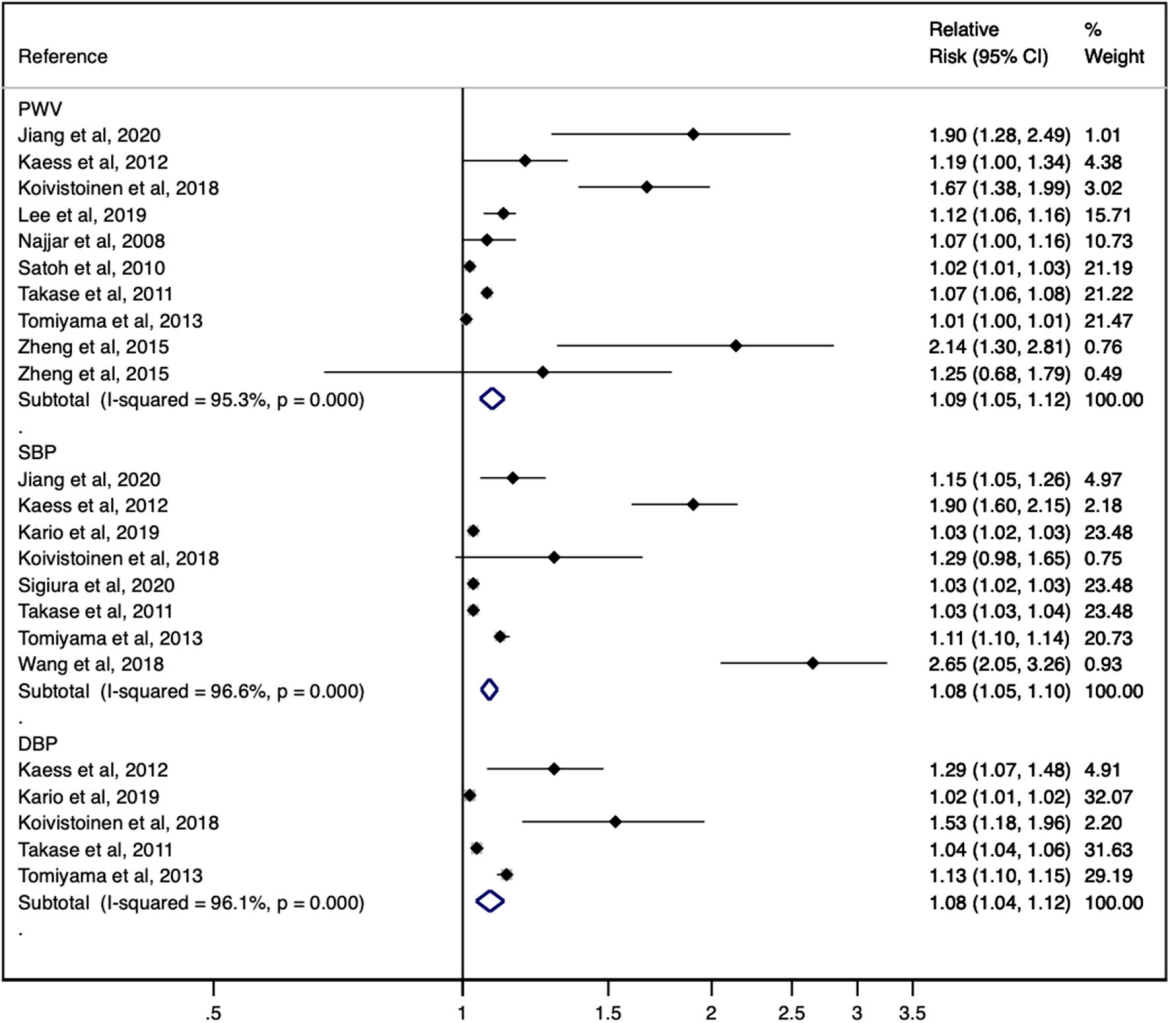
Forest plot including the relative risk of incident hypertension for pulse wave velocity, systolic blood pressure and diastolic blood pressure.

Higher AS, as measured by PWV, was significantly associated with a higher the pooled risk estimate of incident hypertension (RR: 1.09; 95% CIs: 1.05, 1.12). The heterogeneity of this estimate was considerable (*I*^2^ = 95.3%; *p* = 0.00) (Figure 2).

### Association Between Systolic Blood Pressure and Incident Hypertension

The pooled risk estimate of incident hypertension was significantly associated with a higher baseline SBP (RR: 1.08; 95% CIs: 1.05, 1.10). The heterogeneity of this estimate was considerable (*I*^2^ = 96.6%; *p* = 0.00) (Figure 2).

### Association Between Diastolic Blood Pressure and Incident Hypertension

The pooled risk estimate of incident hypertension was significantly associated with a higher DBP (RR: 1.08; 95% CIs: 1.04, 1.12). The heterogeneity of this estimate was considerable (*I*^2^ = 96.1%; *p* = 0.00) (Figure 2).

### Predictive Ability of Pulse Wave Velocity and Systolic Blood Pressure for Incident Hypertension

**Figure 3:**
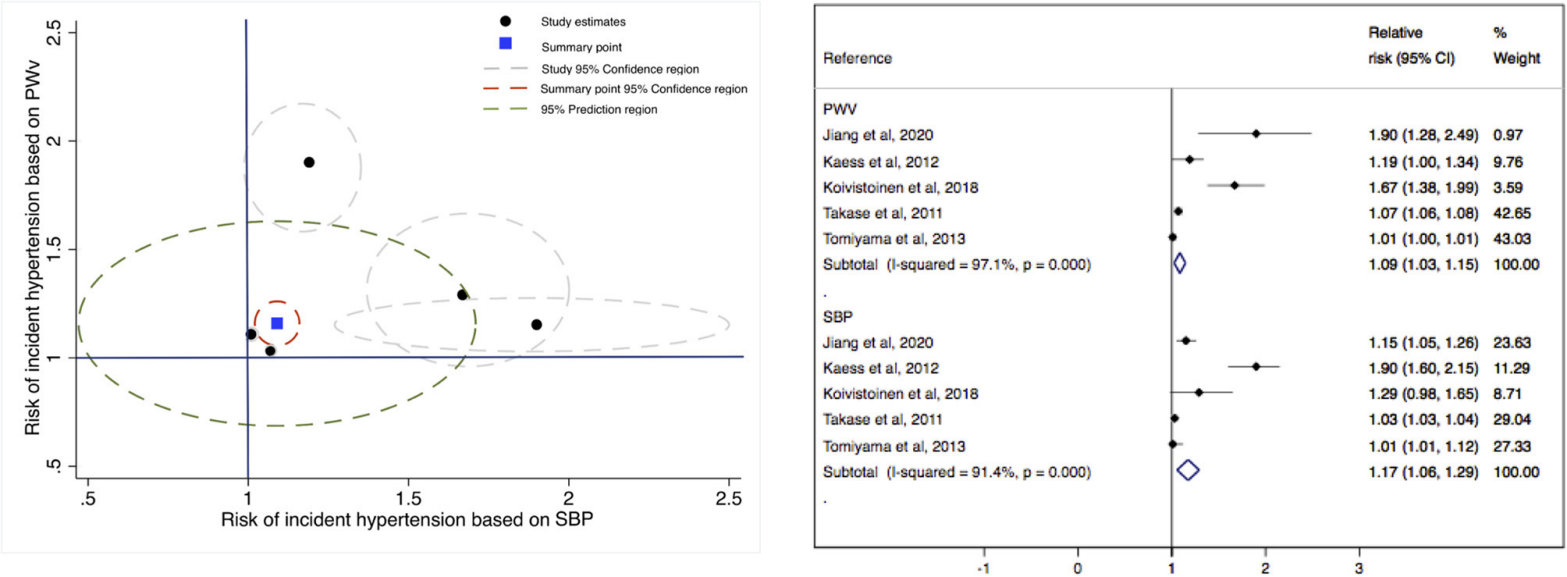
Comprehensive scatterplot for the risk of incident hypertension based on systolic blood pressure (x-axis) and on pulse wave velocity (y-axis).

When a predictive model plot was used to estimate the risk of incident hypertension in the studies with two markers (PWV and SBP), the RR of PWV was 1.09 (95% CIs: 1.03, 1.15), and the RR of SBP for incident hypertension was 1.17 (95% CIs: 1.06, 1.29). The heterogeneity of the two estimates was considered to be considerable (*I*^2^ = 97.1%, *p* = 0.00; and *I*^2^ = 96.1%, *p* = 0.00, respectively) (Figure 3).

### Sensitivity Analysis

The pooled RR estimate for the association between PWV, SBP and DBP with incident hypertension was not significantly modified (in magnitude or direction) when data from individual studies were removed one at a time from the analysis.

### Subgroup Analysis and Meta-Regression Models

When analyses based on PWV type (cf-PWV or ba-PWV) were performed to estimate the risk of incident hypertension, the pooled RR estimates showed significant results for ba-PWV (RR: 1.07; 95% CIs: 1.04–1.10, *I*^2^ = 95.6%) (Supplementary Table 5).

Random-effects meta-regression models showed that mean age, the percentage of women, smoking history and follow-up time were not related to pooled RR estimates (Supplementary Table 6).

### Publication Bias

Finally, evidence of publication bias was found by Egger's test for the estimates of PWV (*p* = 0.046) and SBP (*p* = 0.007), but not for the estimate of DBP (*p* = 0.149) (Supplementary Figures 1–3).

## Discussion

To our knowledge, this is the first systematic review and meta-analysis to evaluate the association between AS and baseline BP in the normotensive range with incident hypertension. Our findings provide a synthesis of the evidence supporting that higher PWV, SBP and DBP are associated with incident hypertension in normotensive adults, with similar independent predictive values. Additionally, our findings highlight the importance of prevention and early detection of this disease, since, when calculating the predictive risk value of incident hypertension in the studies with two markers, a higher PWV increased the risk of incident hypertension by 9%, and SBP increased the risk of incident hypertension by 17%.

According to the evidence, two specific markers of BP, SBP and DBP, are used to define cardiovascular risk factors (20), with hypertension being defined as SBP values of at least 140 mmHg and/or DBP values of at least 90 mmHg (17). In recent years, the BP curve as the sum of MAP (product of cardiac output and total peripheral resistance) and PP (result of intermittent ventricular ejection of the heart) has been considered a predictor of cardiovascular risk (20). Different studies have associated a higher risk of cardiovascular morbidity and mortality in subjects with BP levels in the prehypertension range (SBP of 120 to 139 mmHg and DBP of 80 to 89 mmHg) (3, 38–40), with incident hypertension 3-fold higher during a mean follow-up of 4 years compared to normotensive subjects (38). Another study, according to these results, reported a higher incident hypertension in adults younger than 65 years of age from 5% in normotensive subjects to 37% in subjects with elevated BP and a higher incident hypertension in adults older than 65 years of age from 16 to 50% (41), with older age being associated with a higher risk of incident hypertension (38). Our results support that both SBP and DBP are associated with incident hypertension.

Currently, AS has emerged as an important predictor of cardiovascular events and all-cause mortality (9). Furthermore, the association between AS and higher BP has been described in different studies (22, 42, 43), assuming that the changes occurring in the vascular wall caused by arterial hypertension lead to AS (16, 20, 44). However, recent studies have shown that PWV, considered the gold standard for measuring AS (13), may precede the pathogenesis of arterial hypertension and thus favor the onset of this pathology (2, 16, 18, 19). Our results confirm that higher PWV is associated with incident hypertension. According to the recent ESC/ESH hypertension guidelines, values above 10 m/s have been established for the measurement of cf-PWV as a cardiovascular risk factor in middle-aged and hypertensive patients (17). Another study that established reference values for PWV according to age and BP category supports these results (45). Although the mechanisms linking AS to incident hypertension are unclear, both structural and functional abnormalities of blood vessels have been shown to be related to the subsequent development of arterial hypertension in prehypertensive subjects (46). In addition, several studies have indicated that elastin alterations occurring at the level of the aortic wall, which increase AS, are associated with the development of arterial hypertension (16). In this study, the association between PWV, SBP and DBP with incident hypertension observed in each study was consistently confirmed in the results with all studies combined. Furthermore, although considerable heterogeneity (95.3, 96.6, and 96.1%, respectively) was observed across studies, all studies individually indicated higher PWV, SBP and DBP for association with incident hypertension (concordant heterogeneity).

Given that most of the studies included in the meta-analysis use ba-PWV as the method for assessing AS, and that, so far, cf-PWV is the measure considered the gold standard in AS measurement (13), it seems necessary to establish the potential differences between these two measures. Cf-PWV has traditionally been used as a standard method for the assessment of vascular damage and the prediction of cardiovascular events (13, 17); however, different recent studies have demonstrated that ba-PWV is a valid and effective method for the assessment of vascular damage and the prediction of cardiovascular events (14, 15, 26, 47), highlighting that it does not require any specialized measurement technique since it uses a non-invasive and easy-to-use oscillometric technique for the assessment of AS in daily clinical practice (48, 49). In addition, this technique can provide 24-h ambulatory BP monitoring and PWV estimates (50). Tonometric measurements of cf-PWV are affected by different factors, such as (i) the need for sophisticated equipment, (ii) the need for trained personnel, (iii) the time to perform the procedure, (iv) the possibility of biases in relation to the patient's position, and (v) the possibility of biases in relation to the calculation of the distance between the two measurement points (51–53). This evidence could be relevant in clinical practice, as the measurement of AS may provide information on future diseases, including hypertension (11).

There are some limitations of this study that should be acknowledged. First, most of the included studies showed a moderate or high risk of heterogeneity; therefore, our results should be interpreted with caution. Second, there was evidence of publication bias using Egger's test for PWV and SBP, and unpublished results could modify the results of this meta-analysis. Third, because PWV has been considered as the accepted gold standard for the non-invasive measurement of AS (13), only studies using PWV were included in this systematic review and meta-analysis. Fourth, the scarcity of included studies examining the association between arterial stiffness and BP progression with incident hypertension is noteworthy, and this could affect the association between risk factors such as mean age, percentage of women, and smoking history with incident hypertension. Therefore, prospective longitudinal studies of high methodological quality with large samples testing these findings in populations with different characteristics are needed to further elucidate the association between AS, SBP and DBP with incident hypertension.

## Conclusions

Our results provide a synthesis of the evidence supporting that higher PWV, SBP, and DBP are associated with the development of arterial hypertension. These findings are of clinical importance, considering AS as an additional tool for the prevention of arterial hypertension, highlighting the prevention of this disease as a fundamental component in the reduction of cardiovascular morbidity and mortality.

## Data Availability Statement

The original contributions presented in the study are included in the article/Supplementary Material, further inquiries can be directed to the corresponding author/s.

## Author Contributions

AS-L and IC-R: conceptualization, investigation, and writing—original draft preparation. AS-L, CÁ-B, and IC-R: methodology. IC-R and CÁ-B: software. RB and BN-P: validation and visualization. AS-L and CÁ-B: formal analysis. AS-L, RB, and BN-P: resources. IC-R and VM-V: data curation. VM-V: writing—review and editing. IC-R and RB: supervision. All of the authors revised and approved the final version of the article.

## Funding

This study was funded by the Spanish Ministry of Science and Innovation, Instituto de Salud Carlos III and co-funded by European Union (ERDF/ESF), grant numbers PI21/00008 and RD21/0016/0025. AS-L was supported by a grant from the University of Castilla-La Mancha (2019-PREDUCLM-10708). This article is partly based upon work from COST Action CA18216 VascAgeNet, supported by COST (European Cooperation in Science and Technology, http://www.cost.eu).

## Conflict of Interest

The authors declare that the research was conducted in the absence of any commercial or financial relationships that could be construed as a potential conflict of interest.

## Publisher's Note

All claims expressed in this article are solely those of the authors and do not necessarily represent those of their affiliated organizations, or those of the publisher, the editors and the reviewers. Any product that may be evaluated in this article, or claim that may be made by its manufacturer, is not guaranteed or endorsed by the publisher.
