# Corrigendum: Association Between Arterial Stiffness and Blood Pressure Progression With Incident Hypertension: A Systematic Review and Meta-Analysis

**DOI:** 10.3389/fcvm.2022.877296

**Published:** 2022-03-14

**Authors:** Alicia Saz-Lara, Rosa María Bruno, Iván Cavero-Redondo, Celia Álvarez-Bueno, Blanca Notario-Pacheco, Vicente Martínez-Vizcaíno

**Affiliations:** ^1^Universidad de Castilla-La Mancha, Health and Social Research Center, Cuenca, Spain; ^2^INSERM U970, Paris Cardiovascular Research Centre-PARCC, Université de Paris, Paris, France; ^3^Pharmacology Unit, Hôpital Européen Georges Pompidou, AP-HP, Paris, France; ^4^Rehabilitation in Health Research Center (CIRES), Universidad de las Américas, Santiago, Chile; ^5^Universidad Politécnica y Artística del Paraguay, Asunción, Paraguay; ^6^Universidad Autónoma de Chile, Facultad de Ciencias de la Salud, Talca, Chile

**Keywords:** incident hypertension, arterial stiffness, pulse wave velocity, systolic blood pressure, diastolic blood pressure

In the original article, we neglected to include the funder: ***“This article is partly based upon work from COST Action CA18216 VascAgeNet, supported by COST (European Cooperation in Science and Technology***, ***http://www.cost.eu******).”***

The authors apologize for this error and state that this does not change the scientific conclusions of the article in any way. The original article has been updated.

## Publisher's Note

All claims expressed in this article are solely those of the authors and do not necessarily represent those of their affiliated organizations, or those of the publisher, the editors and the reviewers. Any product that may be evaluated in this article, or claim that may be made by its manufacturer, is not guaranteed or endorsed by the publisher.

