# Supplementary material for: Association Between Arterial Stiffness and Blood Pressure Progression With Incident Hypertension: A Systematic Review and Meta-Analysis
Source: Front Cardiovasc Med. 2022 Feb 11;9:798934. doi: 10.3389/fcvm.2022.798934 (PMC8873377; doi:10.3389/fcvm.2022.798934)

frontiers

Supplementary Material

Supplementary Table 1. Search strategy for the MEDLINE database.

Normotensive adults<br>**OR**<br>Young adults<br>**OR**<br>Older adults<br>**OR**<br>Elderly adults	**AND**	Arterial stiffness<br>**OR**<br>Pulse wave velocity<br>**OR**<br>PWV<br>**OR**<br>Aortic stiffness<br>**OR**<br>Blood pressure<br>**OR**<br>Systolic blood pressure<br>**OR**<br>SBP<br>**OR**<br>Diastolic blood pressure<br>**OR**<br>DBP	**AND**	Onset hypertension<br>**OR**<br>Development hypertension<br>**OR**<br>Incident hypertension

Supplementary Table 2. Quality assessment with the tool for observational cohort and cross-sectional studies of the National Heart, Lung and Blood Institute for pulse wave velocity as a predictor of incident hypertension.

Reference	1	2	3	4	5	6	7	8	9	10	11	12	13	14	Quality
Najjar et al, 2008	Y	Y	Y	Y	Y	Y	Y	Y	Y	Y	Y	NR	N	Y	Good
Satoh et al, 2010	Y	Y	Y	Y	Y	Y	N	Y	Y	N	N	NR	Y	Y	Fair
Takase et al, 2011	Y	Y	N	Y	Y	Y	N	Y	Y	Y	Y	NR	N	Y	Fair
Kaess et al, 2012	Y	N	NR	Y	Y	Y	Y	Y	Y	N	N	NR	NR	Y	Poor
Tomiyama et al, 2013	Y	Y	N	Y	Y	Y	N	Y	Y	Y	Y	NR	N	Y	Fair
Zheng et al, 2015	Y	Y	Y	Y	Y	Y	N	Y	Y	N	N	NR	N	Y	Fair
Koivistoinen et al, 2018	Y	Y	Y	Y	Y	Y	N	Y	Y	N	Y	NR	Y	Y	Good
Lee et al, 2019	Y	Y	Y	Y	Y	Y	N	Y	Y	N	Y	NR	Y	Y	Good
Jiang et al, 2020	Y	Y	Y	Y	Y	Y	N	Y	Y	N	Y	NR	NR	Y	Fair

1. Was the research question or objective in this paper clearly stated?; 2. Was the study population clearly specified and defined?; 3. Was the participation rate of eligible persons at least 50%?; 4. Were all the subjects selected or recruited from the same or similar populations (including the same time period)? Were inclusion and exclusion criteria for being in the study prespecified and applied uniformly to all participants?; 5. Was a sample size justification, power description, or variance and effect estimates provided?; 6. For the analyses in this paper, were the exposure(s) of interest measured prior to the outcome(s) being measured?; 7. Was the timeframe sufficient so that one could reasonably expect to see an association between exposure and outcome if it existed?; 8. For exposures that can vary in amount or level, did the study examine different levels of the exposure as related to the outcome (e.g., categories of exposure, or exposure measured as continuous variable)?; 9. Were the exposure measures (independent variables) clearly defined, valid, reliable, and implemented consistently across all study participants?; 10. Was the exposure(s) assessed more than once over time?; 11. Were the outcome measures (dependent variables) clearly defined, valid, reliable, and implemented consistently across all study participants?; 12. Were the outcome assessors blinded to the exposure status of participants?; 13. Was loss to follow-up after baseline 20% or less?; 14. Were key potential confounding variables measured and adjusted statistically for their impact on the relationship between exposure(s) and outcome(s)?; N: no; NR: not reported; Y: yes.

Supplementary Table 3. Quality assessment with the tool for observational cohort and cross-sectional studies of the National Heart, Lung and Blood Institute for systolic blood pressure as a predictor of incident hypertension.

**Reference**	**1**	**2**	**3**	**4**	**5**	**6**	**7**	**8**	**9**	**10**	**11**	**12**	**13**	**14**	**Quality**
Takase et al, 2011	Y	Y	N	Y	Y	Y	N	Y	Y	Y	Y	NR	N	Y	Fair
Kaess et al, 2012	Y	N	NR	Y	Y	Y	Y	Y	Y	N	N	NR	NR	Y	Poor
Tomiyama et al, 2013	Y	Y	N	Y	Y	Y	N	Y	Y	Y	Y	NR	N	Y	Fair
Koivistoinen et al, 2018	Y	Y	Y	Y	Y	Y	N	Y	Y	N	Y	NR	Y	Y	Good
Wang et al, 2018	Y	Y	NR	Y	Y	Y	N	Y	Y	N	Y	NR	NR	Y	Fair
Kario et al, 2019	Y	Y	Y	Y	Y	Y	Y	Y	Y	N	N	NR	NR	Y	Fair
Jiang et al, 2020	Y	Y	Y	Y	Y	Y	N	Y	Y	N	Y	NR	NR	Y	Fair
Sigiura et al, 2020	Y	Y	Y	Y	Y	Y	N	Y	Y	N	Y	NR	NR	Y	Fair

1. Was the research question or objective in this paper clearly stated?; 2. Was the study population clearly specified and defined?; 3. Was the participation rate of eligible persons at least 50%?; 4. Were all the subjects selected or recruited from the same or similar populations (including the same time period)? Were inclusion and exclusion criteria for being in the study prespecified and applied uniformly to all participants?; 5. Was a sample size justification, power description, or variance and effect estimates provided?; 6. For the analyses in this paper, were the exposure(s) of interest measured prior to the outcome(s) being measured?; 7. Was the timeframe sufficient so that one could reasonably expect to see an association between exposure and outcome if it existed?; 8. For exposures that can vary in amount or level, did the study examine different levels of the exposure as related to the outcome (e.g., categories of exposure, or exposure measured as continuous variable)?; 9. Were the exposure measures (independent variables) clearly defined, valid, reliable, and implemented consistently across all study participants?; 10. Was the exposure(s) assessed more than once over time?; 11. Were the outcome measures (dependent variables) clearly defined, valid, reliable, and implemented consistently across all study participants?; 12. Were the outcome assessors blinded to the exposure status of participants?; 13. Was loss to follow-up after baseline 20% or less?; 14. Were key potential confounding variables measured and adjusted statistically for their impact on the relationship between exposure(s) and outcome(s)?; N: no; NR: not reported; Y: yes.

Supplementary Table 4. Quality assessment with the tool for observational cohort and cross-sectional studies of the National Heart, Lung and Blood Institute for diastolic blood pressure as a predictor of incident hypertension.

Reference	1	2	3	4	5	6	7	8	9	10	11	12	13	14	Quality
Takase et al, 2011	Y	Y	N	Y	Y	Y	N	Y	Y	Y	Y	NR	N	Y	Fair
Kaess et al, 2012	Y	N	NR	Y	Y	Y	Y	Y	Y	N	N	NR	NR	Y	Poor
Tomiyama et al, 2013	Y	Y	N	Y	Y	Y	N	Y	Y	Y	Y	NR	N	Y	Fair
Koivistoinen et al, 2018	Y	Y	Y	Y	Y	Y	N	Y	Y	N	Y	NR	Y	Y	Good
Kario et al, 2019	Y	Y	Y	Y	Y	Y	Y	Y	Y	N	N	NR	NR	Y	Fair

1. Was the research question or objective in this paper clearly stated?; 2. Was the study population clearly specified and defined?; 3. Was the participation rate of eligible persons at least 50%?; 4. Were all the subjects selected or recruited from the same or similar populations (including the same time period)? Were inclusion and exclusion criteria for being in the study prespecified and applied uniformly to all participants?; 5. Was a sample size justification, power description, or variance and effect estimates provided?; 6. For the analyses in this paper, were the exposure(s) of interest measured prior to the outcome(s) being measured?; 7. Was the timeframe sufficient so that one could reasonably expect to see an association between exposure and outcome if it existed?; 8. For exposures that can vary in amount or level, did the study examine different levels of the exposure as related to the outcome (e.g., categories of exposure, or exposure measured as continuous variable)?; 9. Were the exposure measures (independent variables) clearly defined, valid, reliable, and implemented consistently across all study participants?; 10. Was the exposure(s) assessed more than once over time?; 11. Were the outcome measures (dependent variables) clearly defined, valid, reliable, and implemented consistently across all study participants?; 12. Were the outcome assessors blinded to the exposure status of participants?; 13. Was loss to follow-up after baseline 20% or less?; 14. Were key potential confounding variables measured and adjusted statistically for their impact on the relationship between exposure(s) and outcome(s)?; N: no; NR: not reported; Y: yes.

Supplementary Table 5. Subgroup analysis according to the type of pulse wave velocity (cf-PWV or ba-PWV) for the risk of incident hypertension.

	**No. studies (samples)**	**ES (95%CIs)**	**I^2**
**cf-PWV**	2 (2)	1.11 (1.00, 1.21)	36.2%
**ba-PWV**	7 (8)	**1.07 (1.04, 1.10)**	95.6%

Supplementary Table 6. Meta-regression according to mean age, percentage of female, smoking history and follow-up time by type of exposure for the risk of incident hypertension.

	**Coefficient**	**95%CIs**	**P value**
**Pulse Wave Velocity**			
Mean age	-0.001	-0.034, 0.034	0.993
% Women	0.006	-0.002, 0.014	0.115
Smoking history	-0.009	-0.025, 0.007	0.225
Follow-up time	-0.076	-0.348, 0.195	0.538
**Systolic Blood Pressure**			
Mean age	0.030	-0.040, 0.090	0.388
% Women	0.010	-0.012, 0.032	0.304
Smoking history	-0.045	-0.124, 0.033	0.208
Follow-up time	-0.070	-0.276, 0.131	0.417
**Diastolic Blood Pressure**			
Mean age	-0.003	-0.038, 0.033	0.815
% Women	-0.002	-0.004, 0.001	0.068
Smoking history	-0.012	-0.048, 0.025	0.389
Follow-up time	-0.023	-0.112, 0.066	0.474

Supplementary Figure 1. Funnel plot for pooled relative risk of pulse wave velocity.

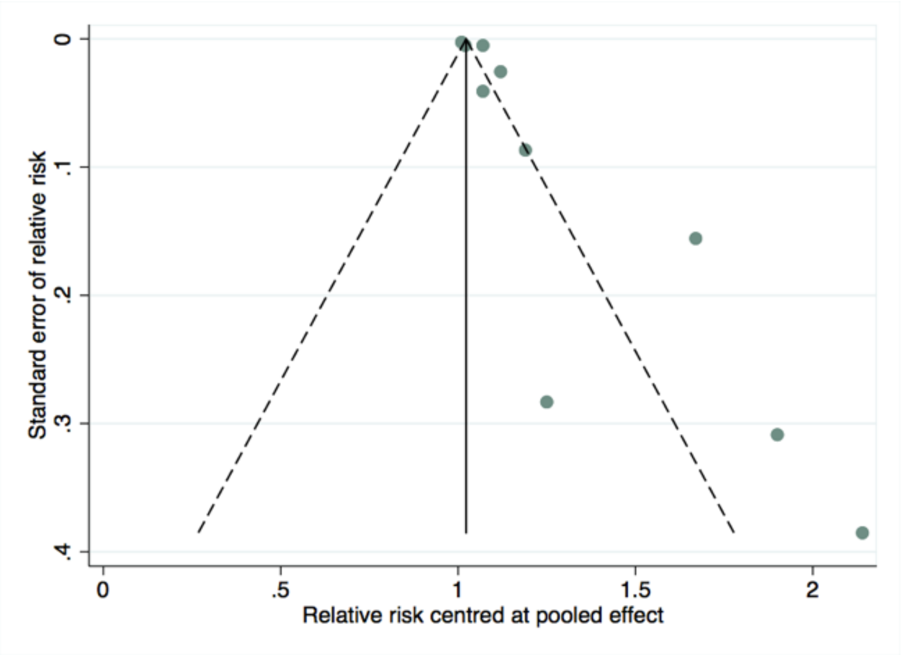

Supplementary Figure 2. Funnel plot for pooled relative risk of systolic blood pressure.

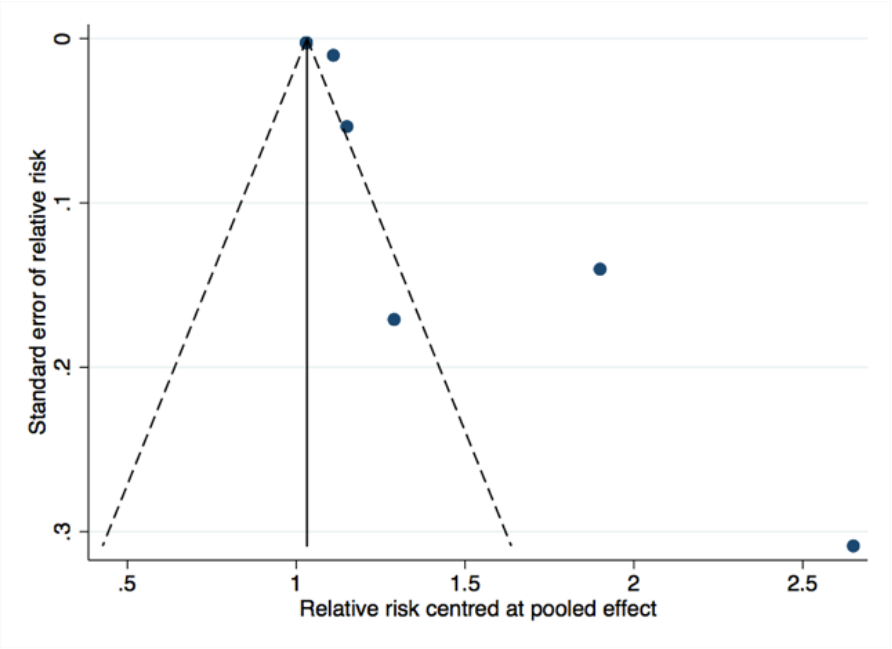

Supplementary Figure 3. Funnel plot for pooled relative risk of diastolic blood pressure.

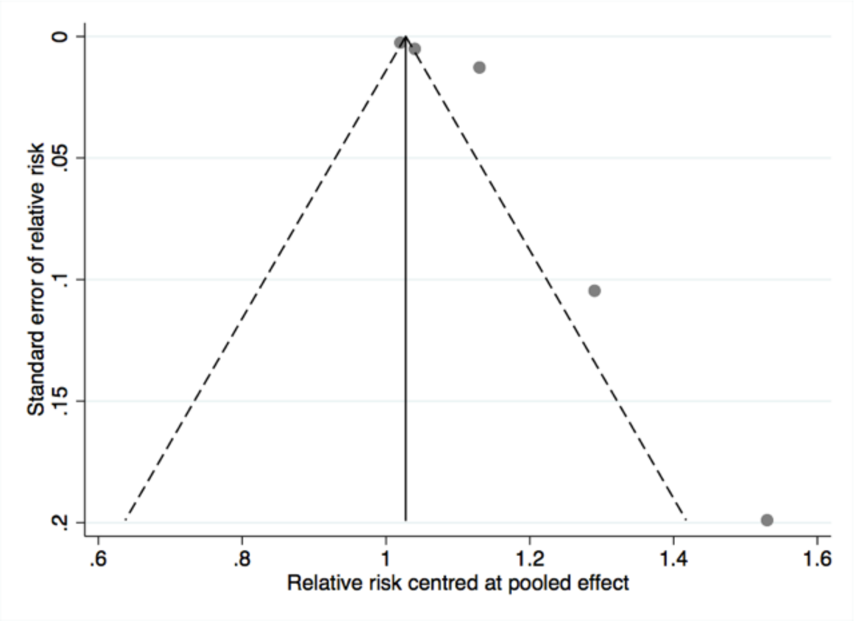

Supplement: Supplementary file 1 [file Data_Sheet_1.PDF]
